# A recall-by-genotype study of *CHRNA5-A3-B4* genotype, cotinine and smoking topography: study protocol

**DOI:** 10.1186/1471-2350-15-13

**Published:** 2014-01-22

**Authors:** Jennifer J Ware, Nic Timpson, George Davey Smith, Marcus R Munafò

**Affiliations:** 1 MRC Integrative Epidemiology Unit, University of Bristol, Bristol, UK; 2 School of Social and Community Medicine, Oakfield House, Oakfield Grove, Bristol BS8 2BN, UK; 3 School of Experimental Psychology, University of Bristol, 12a Priory Road, Bristol BS8 1TU, UK; 4 UK Centre for Tobacco and Alcohol Studies, University of Bristol, Bristol, United Kingdom

**Keywords:** Smoking, Cotinine, Genetics, *CHRNA3*, *CHRNA5*, Smoking topography

## Abstract

**Background:**

Genome-wide association studies have revealed an association between several loci in the nicotinic acetylcholine receptor gene cluster *CHRNA5-A3-B4* and daily cigarette consumption. Recent studies have sought to refine this phenotype, and have shown that a locus within this cluster, marked primarily by rs1051730 and rs16969968, is also associated with levels of cotinine, the primary metabolite of nicotine. This association remains after adjustment for self-reported smoking, which suggests that even amongst people who smoke the same *number* of cigarettes there is still genetically-influenced variation in nicotine consumption. This is likely to be due to differences in smoking topography, that is, how a cigarette is smoked (e.g., volume of smoke inhaled per puff, number of puffs taken per cigarette). The aim of this study is to determine potential mediation of the relationship between the rs1051730 locus and cotinine levels by smoking topography.

**Methods/Design:**

Adopting a recall-by-genotype design, we will recruit 200 adults from the Avon Longitudinal Study of Parents and Children on the basis of minor or major homozygote status at rs1051730 (100 in each genotype group). All participants will be current, daily smokers. Our primary study outcome measures will be measures of smoking topography: total volume of smoke (ml) inhaled per cigarette, total volume of smoke (ml) inhaled over of the course of one day, and salivary cotinine level (ng/ml).

**Discussion:**

This study will extend our understanding of the biological basis of inter-individual variability in heaviness of smoking, and therefore in exposure to smoking-related toxins. The novel recall-by-genotype approach we will use is efficient, maximising statistical power, and enables the collection of extremely precise phenotypic data that are impractical to collect in a larger sample. The methods described within this protocol also hold the potential for wider application in the field of molecular genetics.

## Background

Twin and adoption studies have provided consistent evidence that genetic factors contribute to the aetiology of cigarette smoking, including smoking initiation, progression to heavy use and persistence [1-5]. Advances in the identification of *specific* genetic variants associated with these phenotypes are now being made, principally through the application of genome-wide technologies. Genome-wide association (GWA) consortia comprising multiple cohorts/studies, and large sample sizes (often in excess of 50,000), are becoming increasingly common, with large sample sizes offering increased power to detect the small genetic effects common in complex diseases. Requiring no *a priori* hypotheses, these studies have been successful in determining novel variants associated with disease, including smoking-related behaviours [6].

GWA studies have provided robust evidence of an association between several loci in the nicotinic acetylcholine receptor gene cluster *CHRNA5-A3-B4* (located on the long arm of chromosome 15) and heaviness of smoking [6-8]. One locus within this gene cluster, marked principally by variants rs1051730 in *CHRNA3* and rs16969968 in *CHRNA5* (which are almost perfectly correlated in European populations and therefore essentially interchangeable), has generated particular interest, and has led to renewed interest in these gene products. Research using knock-out mouse models suggests that that locus influences self-titrated nicotine exposure via effects at receptors which influence toxicity of high doses of nicotine [9].

The GWA studies described above employed relatively crude measures of smoking heaviness, namely self-reported daily cigarette consumption. This is often necessary given the need to harmonise phenotype definitions across studies. Objective, precise measures of heaviness of smoking (e.g., levels of cotinine, the primary metabolite of nicotine, or other tobacco metabolites) show a much stronger association with rs1051730 than measures of self-reported heaviness of smoking [10,11]. Munafò and colleagues [12] recently showed a much stronger association of rs1051730 with cotinine level compared to self-reported cigarette consumption. They also showed that the association with cotinine is robust to adjustment for self-reported daily cigarette consumption. This suggests that even among *equal* cigarette consumers there is genetically influenced variation in total nicotine exposure. Munafò and colleagues [12] argue that this is likely to be due to differences in smoking topography, that is, how a cigarette is smoked (number of puffs taken per cigarette, volume of smoke inhaled per puff, and so on).

It is now well-established that smokers modify their smoking behaviour to self-titrate circulating nicotine to a level appropriate to their need [13,14]. Here we present a protocol to determine potential mediation of the relationship between rs1051730/rs16969968 and cotinine levels by smoking topography. The results of this study will determine whether the stronger association observed between this variant and cotinine levels (compared to daily cigarette consumption) is mediated via self-regulation of nicotine exposure. This study will extend our understanding of the biological underpinnings of inter-individual variability in heaviness of smoking, and resulting exposure to smoking related toxins.

## Methods/Design

### Study design

A recall-by-genotype design will be employed, whereby a genetic variant delivering functional change (in this case rs1051730/rs16969968) is used to select participants for further assessment of detailed, clinically-relevant, phenotypes.

### Participants and recruitment

We will prospectively recruit 200 mothers and children from the Avon Longitudinal Study of Parents and Children (ALSPAC; http://www.bristol.ac.uk/alspac/) on the basis of minor or major homozygote status at rs1051730 (100 in each genotype group). All participants will be current, daily smokers (of manufactured cigarettes), in good health. Smoking status will be confirmed during initial screening by a carbon monoxide (CO) breath reading. If this reading is below 10 ppm then a urinary cotinine assessment (yielding a positive or negative result) will also be used to determine smoking status. Participants will be reimbursed for their time with £50 worth of shopping vouchers on completion of the study.

### Inclusion criteria

•Aged 21 years or over;

•Current, daily smoker (of manufactured cigarettes);

•Minor or major homozygote status at rs1051730/rs16969968;

•In good physical and mental health;

•Able to give informed consent as judged by the investigator.

### Exclusion criteria

•Non-smoker;

•Current substance dependence (other than nicotine and caffeine);

•Significant current or past illness;

•Currently pregnant or lactating.

### Ethical considerations and informed consent

Full ethics approval for this study was granted by the ALSPAC Ethics and Law Committee. All potential participants will be issued with an information sheet prior to commencing the study, detailing the purpose and nature of the study. They will also be given the opportunity to raise any questions with the investigators prior to making a decision to participate. Participants will be informed that they are free to withdraw from the study at any time.

### Sample size determination

Our total sample will consist of 200 participants (100 in each of the two genotype groups). Studies of rs1051730 and heaviness of smoking using cigarettes per day indicate a per-allele effect equivalent to approximately one cigarette per day [15]. Assuming a 10 cigarettes/day smoker, we extrapolate that this will correspond to a 70 ml difference in volume inhaled per cigarette, given an average inhaled volume of 700 ml (SD ~200 ml) based on pilot data. An effect of this magnitude will be detectable with 70% power (α = 0.05) in the present study. Studies of rs1051730 and heaviness of smoking using cotinine level indicate a per-allele effect equivalent to a 24.4 ng/ml increase in serum/plasma cotinine level [12]. An effect of this magnitude will be detectable with 80% power (α = 0.05) in the present study.

### Measures and materials

Smoking topography will be assessed using a smoking topography monitor (CReSS Pocket, Borgwaldt KC, Hamburg, Germany). This is a self-contained, battery-operated device, which measures smoking behaviour, with time and date tags assigned at cigarette insertion/removal, providing a highly quantitative view of cigarette smoking behaviour. Data captured include: puff volume; puff duration; puff flow; puffs per cigarette; inter-puff interval; time to first puff; time to removal; volume per cigarette. Onboard memory is used to store all measures, enabling ambulatory monitoring outside of the laboratory.

Smoking topography will be assessed both in the laboratory and in the participants’ 'natural’ environment over the course of one day. Primary outcome measures for smoking topography will be total volume of tobacco smoke consumed per cigarette (ml) and per day (ml). The cigarette smoked in the laboratory will serve additional purposes. Firstly, it will allow participants the opportunity to become familiar with use of the monitor whilst assistance is available. Secondly, it will allow determination of the impact of a single cigarette smoked under controlled conditions on cardiovascular and affect measures.

Cotinine levels will be assessed from saliva. Saliva samples will be collected using salivettes (Sarstedt, Nümbrecht, Germany). Samples will be centrifuged twice (at 5800 rpm for 15 minutes) within 24 hours of collection to ensure removal of human tissue, frozen (at -30°C) and then sent to ABS Laboratories Ltd. for quantitative analysis of cotinine content.

The Fagerström Test of Nicotine Dependence (FTND) [16] will be used to determine level of nicotine dependence. The Brief Questionnaire of Smoking Urges (QSU-Brief) [17,18] and the Positive and Negative Affect Scale (PANAS) [19] will be administered pre- and post- cigarette smoking in the laboratory to assess craving and affect respectively.

Cardiovascular measures (blood pressure and heart rate) will be assessed using the OMRON M6 blood pressure monitor (OMRON Healthcare, UK). Carbon monoxide levels will be assessed using a PiCO + Smokerlyzer (Bedfont Scientific, UK).

Genotyping of rs1051730 has previously been undertaken by KBioscience Ltd. (http://www.kbioscience.co.uk), who use a proprietary competitive allele specific PCR system (KASPar) for single nucleotide polymorphism analysis.

### Primary outcome measures

•Total volume of smoke inhaled per cigarette (ml), and per day (ml);

•Saliva cotinine level (ng/ml).

### Secondary outcome measures

•Number of cigarettes consumed per day (self-reported and objectively assessed);

•Heart rate and blood pressure (pre- and post-cigarette consumption);

•Craving scores (pre- and post-cigarette consumption);

•Positive and negative affect scores (pre- and post-cigarette consumption).

### Procedure

**Figure 1:**
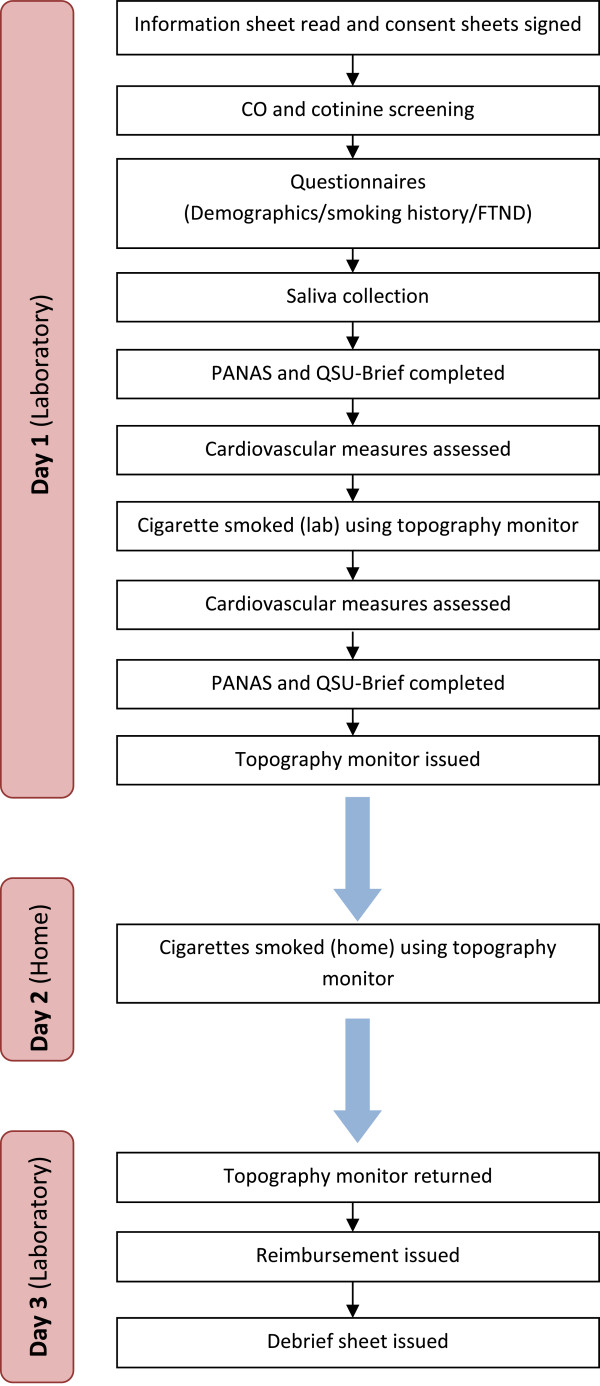
Study procedure.

The study will take place over the course of three days (see Figure 1). On day one participants will attend the research centre for approximately 45 minutes. An information sheet will be provided, and written, informed consent obtained. Smoking status will be confirmed by a CO reading and/or urinary cotinine assessment, as described. Questionnaires will then be used to obtain demographic information, smoking history, and level of nicotine dependence. A saliva sample will then be collected for quantitative assessment of cotinine level. Participants will next be introduced to the smoking topography monitor, and issued with instructions regarding its use. Pre-cigarette PANAS and QSU-Brief questionnaires will be completed, and cardiovascular measures assessed (blood pressure and heart rate). Participants will then be asked to smoke one of their own cigarettes using the smoking topography monitor. This will take place in a ventilated cubicle, under observation by the investigator through one-way glass. The participant will be able to speak to the investigator via an intercom system during the procedure. Post-cigarette PANAS and QSU-Brief questionnaires will then be completed, and cardiovascular measures again assessed. At the end of this session, the participant will be issued with a smoking topography monitor, alongside an information sheet regarding its use and care. On day two, participants will use the smoking topography monitor for each cigarette consumed that day in their natural environment. On day three, participants will return to the research centre to return the device or, if more convenient, the researcher will visit the participant’s home address to collect it. Following this, participants will be reimbursed and a debrief sheet outlining the aims of the study will be issued.

### Statistical plan

Firstly, we will examine the effect of rs1051730 genotype on cotinine level, seeking to confirm the previously observed relationship. Secondly, we will examine the effect of rs1051730 genotype on smoking topography outcome measures (namely volume of smoke inhaled per cigarette and per day). Finally, we will examine the effect of rs1051730 genotype on cotinine level whilst adjusting for smoking topography outcome measures. This will be contrasted with the same association adjusted for self-reported daily cigarette consumption. Multiple regression will be used in all instances, including age and sex as covariates in a forced entry model.

## Discussion

Here we describe a study to investigate potential mediation of the relationship between a locus in the *CHRNA5-A3-B4* gene cluster (rs1051730/rs16969968) and salivary cotinine levels by smoking topography. This study directly builds upon previous research which has shown that this locus is associated with objective measures of heaviness of smoking (cotinine level), even amongst equivalent cigarette consumers based on self-report measures [12]. It will extend our understanding of the biological basis of inter-individual variability in heaviness of smoking, and therefore in exposure to smoking-related toxins.

The study employs a recall-by-genotype design, whereby a genetic variant delivering functional change (in this case rs1051730/rs16969968) is used to select participants (or their biological samples) for further detailed, clinically-relevant, phenotype examination. This novel, innovative approach is warranted for two main reasons. Firstly, it is efficient. In situations where it is necessary to measure expensive phenotypes in order to clarify the nature of a genetic association, statistical power can be improved by using genotype-specific recall instead of random sampling. For example, it is far more powerful to selectively phenotype 100 minor homozygotes and 100 major homozygotes (at opposite ends of a biological gradient) than to phenotype 200 individuals selected at random, because the latter design would recruit far fewer minor homozygotes. Only 16% of Europeans are minor homozygotes at rs1051730 (http://hapmap.ncbi.nlm.nih.gov/), so in an unselected sample of 200 individuals we would expect only 32 minor homozygotes. Secondly, recall-by-genotype studies enable the collection of extremely precise phenotypic data that would be impractical to collect in a much larger sample, given time and expense constraints. Not only do precisely-assessed phenotypes potentially afford increased statistical efficiency, they also afford insight into underlying mechanisms of association.

The recall-by-genotype approach described within this protocol holds the potential for wider application in the field of molecular genetics. This approach could prove particularly useful in the intensive physiological phenotyping of variants found to be related to disease outcomes, serving as a useful follow up strategy for GWA studies (for example, see [20]).

In summary, we describe a study which will build upon and triangulate previous research, and extend our understanding of the biological basis of inter-individual variability in heaviness of smoking, and therefore in exposure to smoking-related toxins. The novel recall-by-genotype approach we will use is efficient, maximising statistical power and enabling the collection of extremely precise phenotypic data that are impractical to collect in a larger sample. The methods described within this protocol also hold the potential for wider application in the field of molecular genetics.

## Trial status

Recruitment for this study commenced in July 2012. Testing is due to commence in November 2013. This study is expected to run until July 2014.

## Competing interests

The authors declare that they have no competing interests.

## Authors’ contributions

MRM, GDS and NT conceived the study. MRM, NT, and JJW participated in the design of the study. JJW drafted the manuscript. All authors read and approved the final manuscript.

## Pre-publication history

The pre-publication history for this paper can be accessed here:

http://www.biomedcentral.com/1471-2350/15/13/prepub
